# Comparison of *In-Vitro* and *Ex-Vivo* Wound Healing Assays for the Investigation of Diabetic Wound Healing and Demonstration of a Beneficial Effect of a Triterpene Extract

**DOI:** 10.1371/journal.pone.0169028

**Published:** 2017-01-03

**Authors:** Christopher Ueck, Thomas Volksdorf, Pia Houdek, Sabine Vidal-y-Sy, Susanne Sehner, Bernhard Ellinger, Ralf Lobmann, Axel Larena-Avellaneda, Konrad Reinshagen, Ina Ridderbusch, Klaas Kohrmeyer, Ingrid Moll, Rolf Daniels, Philipp Werner, Irmgard Merfort, Johanna M. Brandner

**Affiliations:** 1 University Medical Center Hamburg-Eppendorf, Department of Dermatology and Venerology, Hamburg, Germany; 2 University Medical Center Hamburg-Eppendorf, Institute of Medical Biometry and Epidemiology, Hamburg, Germany; 3 Fraunhofer Institute for Molecular Biology and Applied Ecology IME, ScreeningPort, Hamburg, Germany; 4 Klinikum Stuttgart, Department for Endocrinology, Diabetology and Geriatrics, Stuttgart, Germany; 5 University Medical Center Hamburg-Eppendorf, Department of Vascular Medicine, Hamburg, Germany; 6 University Medical Center Hamburg-Eppendorf, Department and Clinic of Pediatric Surgery, Hamburg, Germany; 7 Eberhard Karls University Tübingen, Department of Pharmaceutical Technology, Tübingen, Germany; 8 Albert-Ludwigs-University Freiburg, Department of Pharmaceutical Biology and Biotechnology, Freiburg, Germany; Children's Hospital Boston, UNITED STATES

## Abstract

Diabetes mellitus is a frequent cause for chronic, difficult-to-treat wounds. New therapies for diabetic wounds are urgently needed and *in-vitro* or *ex-vivo* test systems are essential for the initial identification of new active molecules. The aim of this study is to compare *in-vitro* and *ex-vivo* test systems for their usability for early drug screening and to investigate the efficacy of a birch bark triterpene extract (TE) that has been proven *ex-vivo* and clinically to accelerate non-diabetic wound healing (WH), in a diabetic context. We investigated *in-vitro* models for diabetic WH, i.e. scratch assays with human keratinocytes from diabetic donors or cultured under hyperglycaemic conditions and a newly developed porcine *ex-vivo* hyperglycaemic WH model for their potential to mimic delayed diabetic WH and for the influence of TE in these test systems. We show that keratinocytes from diabetic donors often fail to exhibit significantly delayed WH. For cells under hyperglycaemic conditions significant decrease is observed but is influenced by choice of medium and presence of supplements. Also, donor age plays a role. Interestingly, hyperglycaemic effects are mainly hyperosmolaric effects in scratch assays. *Ex-vivo* models under hyperglycaemic conditions show a clear and substantial decrease of WH, and here both glucose and hyperosmolarity effects are involved. Finally, we provide evidence that TE is also beneficial for *ex-vivo* hyperglycaemic WH, resulting in significantly increased length of regenerated epidermis to 188±16% and 183±11% (SEM; p<0.05) compared to controls when using two different TE formulations. In conclusion, our results suggest that microenvironmental influences are important in WH test systems and that therefore the more complex hyperglycaemic *ex-vivo* model is more suitable for early drug screening. Limitations of the *in-vitro* and *ex-vivo* models are discussed. Furthermore our data recommend TE as a promising candidate for *in-vivo* testings in diabetic wounds.

## Introduction

Diabetes mellitus type 2 is an important cause for chronic wounds. Approximately 25% of the patients develop diabetic foot ulcer which is a severe burden for the patients and the society[1]. Therefore, test systems reflecting the diabetic phenotype are needed to investigate the underlying mechanisms and to develop new active pharmaceutical ingredients and formulations for the treatment of these chronic wounds. Animal models, particularly mouse and rat, are often used to investigate diabetic wound healing (WH) and delivered important knowledge [e.g.[2–4]. However, their usability is limited especially in early drug discovery due to ethical and economic aspects. Furthermore, there are physiological differences between rodent and human WH[5]. Pigs are better suited as model system due to physiological similarities to humans [5] but they are even more expensive. To use a human model system and to reduce costs, human *in-vitro* cell culture based systems are often utilized for the investigation of WH. For example, for the investigation of the regeneration phase, *in-vitro* WH assays have been developed using monocultures of human keratinocytes or fibroblasts[6, 7]. These assays allow investigating to which extent changes of migration (in some cases combined with proliferation) influence WH. These more simplified models have the advantage and disadvantage to involve fewer cellular interdependencies which, on one hand, might enable the dissection of the various mechanisms in WH but, on the other hand, makes them less comparable to the *in-vivo* situation. Thus, they are useful for the elucidation of specific aspects of the mode of action, but may be less useful for a general drug screening.

Several variations of these assays exist[6, 7]. When regarding the similarity to the WH process *in-vivo*, the scratch assay is the most comparable, because the removal of cells with a pipette tip wounds the monolayer and results in destroyed and activated cells. The removal of a space holder, which is used in exclusion zone assays, mainly abrogates a growth limitation, and migration in Boyden chambers measures migration of single cells through a pore. Therefore, we focus here on scratch wound assays.

In general, cells of diabetic origin as well as cells treated with high glucose conditions are often used to mimic diabetic conditions [e.g.[8–13]. The first aim of our study was to explore the usability of keratinocytes of diabetic origin or keratinocytes from non-diabetic donors grown under high glucose conditions in scratch wound assays as model systems for the investigation of diabetic WH with respect to early drug screening (end point: scratch wound healing at different time points). High glucose conditions were used in scratch assays before, but contradicting data were published. While some authors [13–15][11–13] described delayed wound closure in keratinocytes under hyperglycaemic conditions, this was not the case in experiments performed by others[14]. To elucidate factors which might lead to these contradicting observations, we performed experiments investigating the influence of the history of cells (juvenile (<5 years) versus adult donors, non-diabetic versus diabetic donors) and microenvironment (different media, presence and absence of supplements) in our *in-vitro* WH assays.

As we observed a dependency on microenvironment, our second aim was to develop a more complex model representing a more physiological context in terms of cells types and subcellular matrix, but still capable of moderate throughput and free of ethical considerations. Thus we developed a porcine ex-vivo WH model under hyperglycaemic conditions and tested whether reepithelialization in these models was delayed similar to diabetic skin and depicted the influence of glucose and hyperosmolarity. Although human and porcine *ex-vivo* skin WH models have successfully been used for the investigation of non-diabetic WH before [e.g. [15–21], the data presented in this study show, to our knowledge, the first successful established diabetic/hyperglycaemic WH model.

Finally, to apply our different test systems and therefore to further investigate their usability, we tested the efficacy of a birch bark extract (triterpene extract, TE) which is known to improve WH under non-diabetic conditions *in-vivo* and *ex-vivo* [22–24] and which is approved by the European Medicines Agency for mid-dermal wounds. We show that the *in-vitro* assays under euglycaemic conditions were not able to reproduce the beneficial effect of TE on WH seen *in-vivo*. Also under hyperglycaemic conditions TE did not improve scratch WH *in-vitro*. However, there was a stimulating effect on reepithelialization in *ex-vivo* WH models under hyperglycaemic conditions, similar to what was shown in *ex-vivo* models and *in-vivo* under euglycaemic conditions[22, 24]

## Materials and Methods

### Tissues

Normal skin tissue samples for keratinocyte cultures from adult donors were derived anonymously during routine clinical removal of epidermal cysts and tumors. The samples were localized at least 2 cm from the respective lesions. Skin samples for juvenile non-diabetic keratinocyte and fibroblast cultures were derived from circumcisions. The utilization of adult and juvenile skin was approved by the ethics committee of the Ärztekammer Hamburg: vote No 060900 and WF-61/12. Juvenile: n = 4, male, age < 5 years; adult: n = 7; 5 male, 1 female, 1 unknown; age 66–93 years, mean 75 years; Tissue samples from diabetic donors were derived from the upper thigh of patients with diabetes type 2 (approved by the ethics committee of the Ärztekammer Hamburg: vote No. PV4198). n = 7; 6 male, 1 female, age 59–86, mean 71 years, HbA1c: 5.5%– 8.8% (37 mmol/mol– 73 mmol/mol); mean 7.1% (54 mmol/mol). All patients (or their next of kin) gave their written informed consent. All investigations were conducted according to the principles expressed in the Declaration of Helsinki. Note that not all donors were included in all experiments (see figure legends for n-numbers).

### Media, extracts and formulations

K-SFM (Gibco/Life Technologies, Darmstadt, Germany), EpiLife (Cascade Biologics; Invitrogen, Darmstadt, Germany), and DermaLife (Lifeline Cell Technology/Cellsystems, Troisdorf, Germany) were used for keratinocyte cultures, RPMI with 10% FCS (both Biochrom, Berlin, Germany) for fibroblast culture. TE (Table 1) and betulin were obtained from Birken AG (Niefern-Öschlbronn, Germany). For *in-vitro* cell culture experiments TE and betulin were dissolved in DMSO and further diluted in medium. For controls, DMSO was diluted in medium with the same dilution factor. Oleogels containing 10% (w/w) TE were prepared by dispersing the TE in sunflower oil using an Ultra-Turrax T25 (IKA, Staufen, Germany) at 8000 rpm for 3 min. After a storage period of 24 h, the predetermined amount of water was added to the oleogels using a syringe-to-syringe mixing technique yielding homogenous w/o-emulsions.

**Table 1:** Chemical composition and physical characteristics of the TE used.

**Chemical composition**	Betulin 86.85%, lupeol 3.94%, betulinic acid 3.54%, erythrodiol 0.77%, oleanolic acid 0.62%, unidentified substances 4.28%
**Specific surface area**	42 ± 0.4 m^2^/g
**Particle size D50%**	5.8 μm

### Cell culture

For cultivation of keratinocytes and fibroblasts from human foreskins and adult tissues see[20].

For euglycaemic conditions, keratinocytes were cultured in the respective keratinocyte medium containing 6 mM glucose, fibroblasts in normal RPMI medium (containing 11 mM glucose), for hyperglycaemic conditions, in medium containing 25 mM glucose (keratinocytes and fibroblasts) as well as 50 mM glucose (fibroblasts) and for hyperosmolaric conditions in medium containing 6 mM glucose and 19 mM mannitol for 48 h prior to the start of the experiment.

### Scratch assays

Conventional and semi-automated (high throughput) (Essen BioScience, Ltd., Hertfordshire, United Kingdom) scratch assays were used. For details, comparison and discussion of the two systems see S1 File, S1 Table and S1 Fig.

### Evaluation of scratch WH progress

The images documenting the WH progress were evaluated by using the software TScratch[25]. Non-wounded areas within a visual field were marked, while the scratch wound was left out. Based on this classification, TScratch calculated the scratch wound area as a percentage of the total visual field. To depict the scratch wound area in mm^2^ a picture of a length scale was taken with the same settings and the measurement tool of ImageJ software (NIH, Bethesda, MD; http://imagej.nih.gov/ij) was used.

### Diabetic porcine *ex-vivo* WH model

Non-diabetic *ex-vivo* WH models were produced as described previously [[26]; patent No DE10317400]. For diabetic conditions, the 6 mm biopsies were incubated with 50 mM glucose for 48 h prior to wounding and also after wounding. For hyperosmolaric conditions, 6 mM glucose and 44 mM mannitol were used. WH was stopped 48 h after wounding by shock freezing and the WH progress was evaluated by measuring the length of the regenerated epidermis in H+E stained sections of the central part of the models with an Axiophot II microscope (Zeiss, Jena, Germany) and the measurement tool of the Openlab software 2.0.9 (Improvision, Coventry, UK).

### Cytokine expression

To investigate the influence of TE on cytokines at different glucose levels in fibroblasts we seeded 50,000 cells per 6-well, incubated for 3 days in RPMI, adjusted then glucose levels to 25 mM and 50 mM, incubated for 24 h in medium including 10% FCS and then another 24 h in medium without 10% FCS. Subsequently we added 1 μg/ml or 10 μg/ml TE respectively, and incubated for another 6 h. DMSO in the concentration as used in the higher concentrated TE preparation was used as control. Cells were then harvested in RTL buffer and mRNA isolation was performed with the Micro RNA kit from Qiagen (Hilden, Germany) according to the manufacturer´s instructions. 1 μg of total RNA was used for first-strand cDNA synthesis with the cDNA synthesis kit from Thermo Fisher Scientific (Waltham, MA, USA) as suggested by the manufacturer. 4.5 μl of a 1:25 dilution of cDNA was used as a template in real-time quantitative PCR analysis with FAM dye-labeled TaqMan probes (GAPDH: Hs03929097_g1; IL-1 ß: Hs01555410_m1; IL-8 Hs00174103_m1; IL-6: Hs00985639_m1; CCL2: Hs002334140_m1; FGF2: Hs00266645_m1; FGF7: Hs00940253_m1; all Invitrogen Darmstadt, Germany) in a Light Cycler 96 (Roche Diagnostics, Mannheim, Germany) under conditions recommended by Roche. All real-time quantitative PCR analyses were performed with n = 4 in technical triplicates. Relative transcriptional levels comparing different treatments were calculated using the 2- ΔΔ Ct method[27].

### Statistical analyses

Data are presented as mean values +/- standard error of mean.

For all settings, except cytokine expression, multilevel mixed-effects linear regressions with setting-dependent random intercepts were performed. The distribution of the data was checked and if necessary log transformed to reach normal distribution.

Using the Likelihood-ratio test the significance of the predictors as well as their interaction was tested. Two-tailed values of p< 0.05 were considered statistically significant. In the case of insignificant effects the predictor/interaction was excluded from the model (backward elimination). For the resulting model polynomial-contrast-tests with Bonferroni adjustment for multiplicity were performed to compare subgroups. The appropriate means with corresponding 95% confidence intervals and p-values were reported, all log transformed values were back-transformed.

For *ex-vivo* models, “wound” was chosen as random intercept to account for the repeated measurement structure within each wound. Interaction between glucose/mannitol concentration and the influencing factor (preincubation and treatment, respectively) were included as fixed effects.

For all *in-vitro* models, the cluster structure of the data was modeled with three random effects. First, the different technical replicates were nested within the appropriate cell donors, which were modeled by two nested random effects. Furthermore all technical replicates were measured at several regions of interests (ROI), therefore additionally a crossed random effect for ROI was included. Across the *in-vitro* settings only the fixed effects/predictors varied and were appropriately included as main effect and interaction term (S2 Table). As outcome the change of the scratch wound size as difference from the initial value was modeled to depict for time effects. To control potential bias the baseline size was included as confounder.

For comparison of cytokine expression levels ANOVA with Bonferoni post hoc test was used.

The analyses were conducted with StataCorp. 2015. Stata Statistical Software: Release 14. College Station, TX: StataCorp LP, or SPSS version 22.

## Results

### Diabetic versus non-diabetic cells

**Fig 1:**
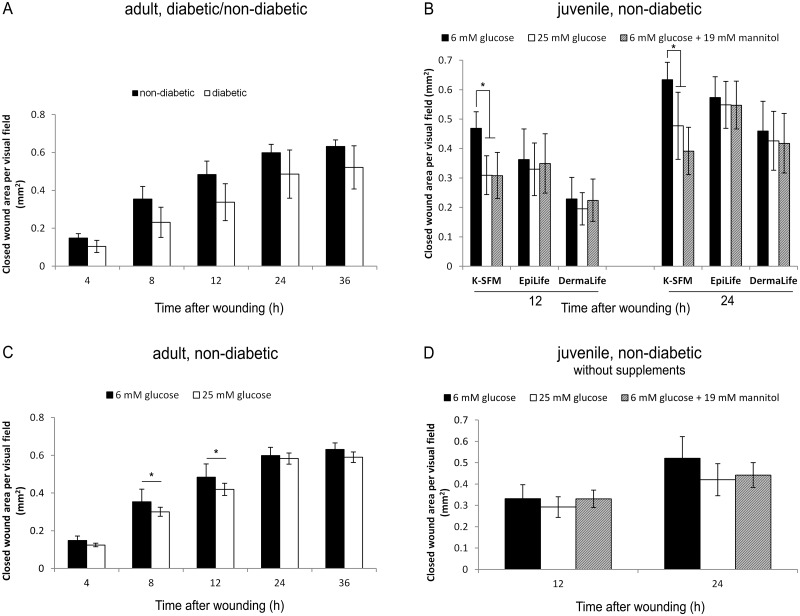
Comparison of scratch wound healing of human primary keratinocytes of various origins and under various culture conditions. Closed wound area per visual field (mm^2^) of (A) diabetic versus non-diabetic keratinocytes at 4, 8, 12, 24 and 36 h after wounding using K-SFM medium. n = 4 in duplicates (non-diabetic keratinocytes) and n = 3 in duplicates (diabetic keratinocytes) (B) Keratinocytes from juvenile donors under euglycaemic (6 mM glucose), hyperglycaemic (25 mM glucose) and hyperosmolar (6 mM glucose + 19 mM mannitol) conditions at 12 and 24 h after wounding in K-SFM, Dermalife and Epilife medium. n = 4 in duplicates. (C) Keratinocytes from adult, non-diabetic donors under euglycaemic (6 mM) and hyperglycaemic (25 mM) conditions 4, 8, 12, 24 and 36 h after wounding in K-SFM medium. n = 4 in duplicates. (D) Keratinocytes from juvenile donors under euglycaemic, hyperglycaemic and hyperosmolar conditions at 12 and 24 h after wounding in K-SFM without supplements. n = 4 in duplicates; mean ± SEM; *: statistically significant with p < 0.05.

At first we tested adult diabetic versus non-diabetic keratinocytes under euglycaemic conditions to investigate the usability of these cells in early drug screening for diabetic wound healing. We observed a trend of decreased scratch wound closure rate for cells of diabetic origin versus non-diabetic origin in conventional scratch assay (Fig 1A); but this was not the case in a high-throughput, semi-automated system (S1A and S1B Fig).

### Non-diabetic cells under hyperglycaemic versus euglycaemic conditions

It was shown by Lan et al. [12, 13] that high glucose conditions result in decreased scratch wound closure in non-diabetic juvenile keratinocytes. We therefore investigated juvenile (foreskin) keratinocytes, as a well understood and frequently applied cellular system, and adult keratinocytes, as a system closer to the real scenario of elderly patients with diabetes mellitus type 2, whether we can recapitulate this finding. We observed that hyperglycaemic conditions significantly (p<0.05) decreased scratch WH in juvenile keratinocytes (Fig 1B; 12 h: closed wound area under euglycaemic conditions: 0.47 ± 0.06 mm^2^, closed wound area under hyperglycaemic conditions: 0.31 ± 0.07 mm^2^, i.e. 66 ± 14% of control; 24 h: closed wound area under euglycaemic conditions: 0.63 ± 0.06 mm^2^, closed wound area under hyperglycaemic conditions: 0.48 ± 0.11 mm^2^, i.e. 68 ± 14% of control) and adult, non-diabetic keratinocytes (Fig 1C; 8 h: euglycaemic conditions: 0.37 ± 0.07 mm^2^, hyperglycaemic conditions: 0.30 ± 0.02 mm^2^, i.e. 85 ± 7% of control; 12 h: euglycaemic conditions: 0.48 ± 0.07 mm^2^, hyperglycaemic conditions: 0.42 ± 0.03 mm^2^, i.e. 87 ± 7% of control). The decrease was also seen with the semi-automated system (S1D Fig). However, of interest, a decrease of wound closure rate was only seen in K-SFM, but not in Dermalife or Epilife medium (Fig 1B). Thus, the choice of medium is of importance. Due to these results, K-SFM medium was chosen for the further experiments. In addition, decrease was seen in juvenile cells at 12 h and 24 h after scratch, but in adult cells only after 12 h, and it was less pronounced (Fig 1B and 1C). Thus, also donor age plays a role.

As the main differences between the media are the supplements added to the basal media, we tested the influence of the supplements present in K-SFM on the hyperglycaemic effect. We observed that while hyperglycaemic conditions resulted in significantly reduced WH in medium containing supplements (Fig 1B), this was not the case in medium without supplements (Fig 1D).

In addition, we investigated whether the high glucose effect can be mimicked by high osmolarity. Indeed, the use of mannitol instead of glucose resulted in similarly delayed wound closure rates in non-diabetic keratinocytes (Fig 1B; 12 h: euglycaemic conditions: 0.47 ± 0.06 mm^2^, hyperosmolaric conditions: 0.31 ± 0.08 mm^2^, i.e. 66 ± 17% of control; 24 h: euglycaemic conditions: 0.63 ± 0.06 mm^2^, hyperosmotic conditions: 0.39 ± 0.08 mm^2^, i.e. 63 ± 15% of control). Again, this effect was only seen in cells cultured with supplements (Fig 1B and 1D).

### Ex-vivo WH model under hyperglycaemic versus euglycaemic conditions

**Fig 2:**
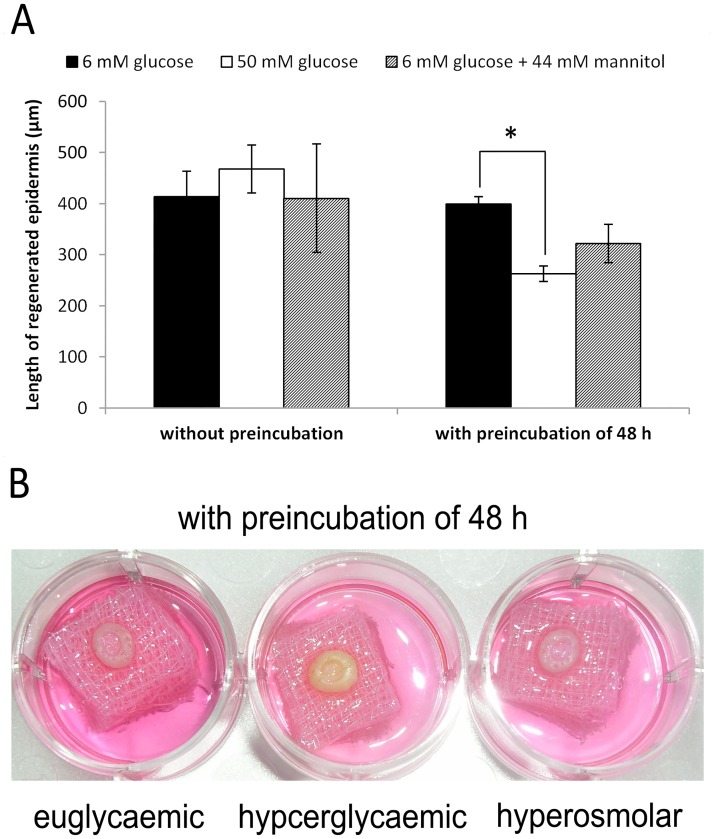
*Ex-vivo* porcine wound healing models cultured under euglycaemic, hyperglycaemic and hyperosmolar conditions. (A) Length of the regenerated epidermis for the experimental settings with and without preincubation for 48 h prior to wounding under euglycaemic (6 mM glucose), hyperglycaemic (50 mM glucose) and hyperosmolar (6 mM glucose + 44 mM mannitol) conditions 48 h after wounding. n = 8 in triplicates; mean ± SEM; *: statistically significant with p < 0.05; (B) Yellow coloring of the skin biopsies cultured under hyperglycaemic conditions for a total of 96 h (48 h preincubation and 48 h after wounding).

As shown above, the choice of medium and the presence of supplements in the medium had a remarkable influence on the effect of hyperglycaemic conditions on the rate of scratch wound closure. In an *in-vivo* wound, these factors may be provided by soluble substances produced by other cell types. Therefore, we established a more complex high glucose porcine *ex-vivo* WH model that contains all essential cells residing in the skin. This model is based on our previously described euglycaemic porcine *ex-vivo* WH model[16, 18, 26]. A significant (p<0.05) decrease in length of regenerated epidermis from 399 ± 15 μm under euglycaemic conditions to 263 ± 15 μm under hyperglycaemic conditions, i.e. to 66 ± 4% of control was observed when using 50 mM glucose conditions and preincubation before wounding the tissue (Fig 2A). This was not the case without preincubation (Fig 2A) or at a 25 mM glucose concentration with or without preincubation (S2 Fig). Mimicking the high osmotic conditions by substituting 44 mM glucose by mannitol resulted also in a decrease of WH in the *ex-vivo* model but to a lesser extent than in the models under hyperglycaemic conditions (to 322 ± 38 μm, i.e. 81 ± 9% of control) and in a non-significant manner (Fig 2A). Of note, high glucose conditions also resulted in a yellowish color of the skin which was not the case in the hyperosmolar models (Fig 2B).

### Influence of TE on *in-vitro* and *ex-vivo* wound healing

Finally, we tested the effect of TE and its main constituent, betulin, on its WH capacity in the eu- and hyperglycaemic and diabetic *in-vitro* and the hyperglycaemic *ex-vivo* assay systems. It was shown before, that this extract, approved by the European Medicines Agency, exhibited a positive effect on non-diabetic *in-vivo* WH [23, 28] as well as on *ex-vivo* WH models under euglycaemic conditions[22, 24].

However, TE did not show a beneficial effect in any of the *in-vitro* tests (juvenile keratinocytes, adult keratinocytes, euglycaemic conditions, hyperglycaemic conditions; see S2 File and S3, S4 and S5 Figs). This was also true for betulin except for a slight but significant scratch WH accelerating effect 24 h after scratch wounding (from 0.50 ± 0.02 mm^2^ to 0.53 ± 0.02 mm^2^, 106 ± 4.4% of control) (see S2 File and S3, S4 and S5 Figs). There was also no beneficial influence of TE on scratch wound healing in fibroblasts (S6 Fig).

**Fig 3:**
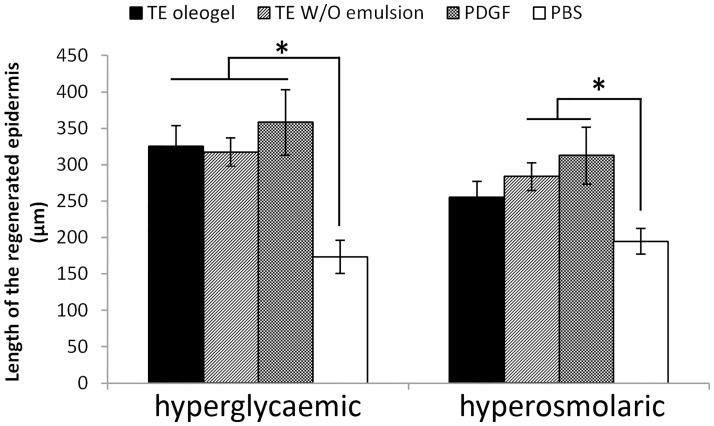
Influence of TE on reepithelialization of hyperglycaemic and hyperosmolaric ex-vivo models. Length of the regenerated epidermis of porcine *ex-vivo* wound healing models that were treated with TE oleogel, W/O emulsion (60%) containing 10% TE oleogel, PDGF (120 ng/ml) or PBS directly after wounding for 48 h under hyperglycaemic or hyperosmolaric conditions (including preincubation); n = 16 in duplicates; mean ± SEM; *: statistically significant with p < 0.05.

The non-existing or even negative effect of TE and betulin on *in-vitro* WH under euglycaemic conditions was surprising because the extract positively influences WH i*n-vivo* and *ex-vivo*[22–24]. Therefore, we concluded that this beneficial effect is dependent on the interplay with other cells. To evaluate whether this might also be the case for diabetic WH, we tested TE in two different formulations in our hyperglycaemic *ex-vivo* model. We used an oleogel identical to the one used *in-vivo / ex-vivo* for non-diabetic wounds [22–24] and a water-in-oil emulsion. The latter was used because it is the basis of a more advanced formulation concept which will finally lead to TE-containing foams, which can be applied to wounds almost touchless. However, the addition of a water phase can influence the availability of the TE and has thus to be studied in addition to the oleogel. Indeed, we observed a significantly accelerating influence of both formulations on epidermal regeneration 48 h after wounding in *ex-vivo* wounds under hyperglycaemic conditions (oleogel: 326 ± 28 μm compared to 173 ± 23 in PBS treated controls, that means 188 ± 16% of control, w/o emulsion: 317 ± 20 μm, that means 183 ± 11% of control) (Fig 3). The positive control, PDGF, which was shown before to be effective in *in-vivo* diabetic wounds [29] also showed a significantly positive effect (358 ± 45 μm, that means 206 ± 26% of control) (Fig 3). There was also a significantly accelerating effect of the TE W/O emulsion (284 ± 19 μm compared to 195 ± 18 μm, that means 146 ± 10% of control) and PDGF (312 ± 39 μm, that means 160 ± 20% of control) on models under hyperosmolaric conditions, but this was less pronounced than for the models under hyperglycaemic conditions (Fig 3). For TE oleogel, no significant effect on models under hyperosmolaric conditions was observed (Fig 3).

### Influence of TE on cytokine expression in fibroblasts

One important difference between the *in-vitro* and the *ex-vivo* test system is the presence of additional cell types which might also influence WH, e.g. via cytokines. As the major second type of cells in the skin is fibroblasts, we tested whether TE might induce the expression of cytokines in fibroblasts under different glucose conditions. We investigated cytokines which have previously been shown to be induced by TE in fibroblasts under normal culture conditions [30] and which are important for wound healing[31]. We observed a significant increase of IL-6, IL-8, IL-1 ß, and CCL-2 expression in fibroblasts under various glucose concentrations (Table 2).

**Table 2:** Influence of TE on cytokine expression under various glucose concentrations (n = 4).

	DMSO control			TE 1 μg/ml			TE 10 μg/ml		
Glucose Conc.	normal	25 mM	50 mM	normal	25 mM	50 mM	normal	25 mM	50 mM
IL-6	0.7	1.3	0.9	13.7	7.8	14.1	143.4	58.4	184.5
				***	***	**	***	***	***
IL-8	2.7	1.8	0.7	152.4	23.6	17.6	1013.3	115.5	75.4
				P = 0.055	***	*	**	***	**
IL-1beta	2.0	1.4	0.8	2.2	5.5	3.5	6.2	5.0	7.0
					*		*	*	**
CCL2	2.5	1.9	1.0	16.9	4.8	4.7	28.1	5.2	7.7*
				*			**		*
FGF2	0.9	1.0	0.7	0.9	1.1	1.0	1.7	1.3	1.3
							***		*
FGF7	0.6	0.7	0.9	0.7	1.1	1.0	1.6	0.8	1.5
							***		

Values denote fold increase/decrease compared to untreated control cells with the same glucose conditions.

*: p<0.05;

** p<0.01;

*** p<0.001 compared to DMSO controls with the same glucose conditions.

## Discussion

### Diabetic keratinocytes as a putative screening system

We did not find significantly decreased *in-vitro* wound closure in cells from diabetic origin, even though *in-vivo* delayed WH is a hallmark of diabetes type 2[32]. This might be due to the high variability of wound closure rates of the diabetic cells and might reflect different adjustment of blood glucose levels and HbA1c values. But also the non-diabetic controls showed a certain level of variability. Thus, cells of diabetic skin, even though valuable because they comprise—at least in early passages [20]—the diabetic history of the donors, may only give reliable results for drug screening when using a high number of donors which is in practice often difficult to achieve due to limited access to donor tissues.

### High glucose conditions decrease *in-vitro* WH but only in a particular medium

While some authors [11–13] observed delayed scratch WH in juvenile keratinocytes cultured under hyperglycaemic conditions, this was not the case in experiments from others [14] even though using the same amount of glucose. However, when looking into detail, the authors used different media. Thus we compared different media and indeed found that high glucose only resulted in delayed WH in K-SFM—which was also used in [11–13]—and that this effect is dependent on the presence of supplements. Unfortunately, the companies are quite restrictive in elucidating the composition of supplements, so we can only speculate about the pivotal substances. For example, the concentration of epidermal growth factor (EGF) is 50 times higher in K-SFM than in Epilife (there is no information for Dermalife). EGF plays an important role during WH because it is necessary for the activation of keratinocyte migration[33]. Furthermore, it was shown that EGF causes a mild hyperglycaemia in mice[34]. In general, supplements reflect substances normally produced by other cell types present in the natural microenvironment of cells. Thus, this dependency on supplements underlines the importance of the interplay of several cell types and their products to mimic the *in-vivo* effect of delayed WH.

### Decreased *in-vitro* WH due to hyperglycaemic conditions can be mimicked by hyperosmolaric conditions

The wound closure delaying effect observed under hyperglycaemic conditions could be a glucose effect, e.g. by the formation of advanced glycation end products (AGEs) [35] or by enhancing glucose metabolism in the cells including increased formation of reactive oxygen species[3, 36]. However, it could also be the result of an osmotic effect. It is known that hyperosmolar conditions can enhance the inflammatory response by the secretion of cytokines[37]. Additionally, hyperosmotic stress might be associated with insulin resistance in diabetes type 2 by inhibiting insulin-mediated signal transduction in adipocytes[38]. To determine the contributing factors, we substituted the elevated amount of glucose by mannitol. Indeed, we could show that the osmotic effect plays a major role. Thus, when using hyperglycaemic keratinocytes in scratch wound assays to mimic diabetic WH for drug screening, one has to keep in mind that mainly the hyperosmolaric aspect of hyperglycaemia plays a role and that only substances that influence the effect of hyperosmolarity will be detected.

### Decreased WH in the *ex-vivo* WH model under hyperglycaemic conditions

The effect of high glucose on 2D keratinocyte cultures was significantly influenced by the microenvironment. The influence of the microenvironment in *in-vivo* wounds is given by other cell types and their interplay with keratinocytes. To better mimic this *in-vivo* situation, we established a porcine *ex-vivo* WH model that provides already intrinsically additional cell types. For this so-called “diabetic” WH model, an elevation to 25 mM glucose—which is used in 2D culture—is not sufficient. This might be due to the fact that glucose reaches the wound closing keratinocytes only via diffusion and thus the final concentration in the epidermis is lower. Of note, fibroblasts, which are the first cells in contact with glucose, are known to grow better with higher concentrations of glucose than 6 mM. Typical culture media for fibroblasts contain either 11.1 mM glucose (RPMI 1640 medium) or even 25 mM glucose (DMEM w/o L-glutamin). Thus, a negative effect on fibroblasts can only be achieved with higher concentrations. It is also necessary to preincubate the model for 48 h, which reflects that glucose needs some time to execute its effect. Of note, decreased WH in the *ex-vivo* model is not completely mimicked by hyperosmolaric conditions, suggesting that also typical glucose effects play a role. This is also supported by the fact that the hyperglycaemic model, but not the hyperosmolaric one, shows a yellowish color which may be due to AGEs as reported by Yokota and Tokudome[39]. Thus, with the *ex-vivo* model a hyperglycaemia-induced delay in WH comprising glucose-chemistry as well as osmolarity effects can be generated reproducibly. This model is based on porcine skin, thus tissue accessibility is not a problem and the model is not regarded as an animal experiment, as tissue from slaughtered animals is used. However, in comparison to *in-vivo* WH this model also has important limitations. There is no blood supply, no new invasion of immune cells and no long-term diabetic effects (e.g. neuropathy) which can only be mimicked by *in-vivo* models.

### Beneficial effect of TE under hyperglycaemic conditions only in *ex-vivo*, but not in *in-vitro* models

As we knew about the beneficial effect of TE *in-vivo* and *ex-vivo* under euglycaemic conditions [22–24] and its effect on cytokine production and cytoskeleton in diabetic keratinocytes and fibroblasts [30] and because triterpenes are known to have beneficial effects in diabetes[40], we used TE as a test substance in our various *in-vitro* and *ex-vivo* models. Interestingly, it only showed a positive effect in the *ex-vivo* model. An explanation for the discrepancy between the *in-vitro* and the *ex-vivo* effects is probably the fact that *ex-vivo* many more cell types are involved and that their interplay may be important. Indeed we could show that fibroblasts under hyperglycaemic conditions release cytokines in response to TE treatment. These cytokines, in turn, may influence other dermal cells important for wound healing, including keratinocytes. But of course the influence of TE is likely not restricted to fibroblasts and keratinocytes, but can include further dermal cells like dermal immune cells or—at least *in-vivo*–immigrating extra-dermic cells[41].

Of note, we observed also under euglycaemic conditions no effect or even a long term negative effect *in-vitro*, in contrast to the positive effect observed *ex-vivo* and *in-vivo*[22–24]. This clearly supports that the *ex-vivo* model reflects better the *in-vivo* situation and is therefore more suitable for early drug screening. Thus, the positive effect of TE in the hyperglycaemic *ex-vivo* system may indeed hint for a beneficial effect of the birch bark extract in *in-vivo* diabetic WH.

Summarizing our data the “hyperglycaemic” *ex-vivo* model turns out to be the more reliable system for a first test of new active ingredients compared to the *in-vitro* scratch assay. Nonetheless, the *in-vitro* systems may also be useful to depict specific influences on isolated cell types and mechanisms.

In addition, one has to keep in mind that hyperglycemic conditions in vitro alone cannot replicate the complex damage that diabetes causes over time *in-vivo*. *In-vitro* assays as well as our porcine *ex-vivo* assay lack "chronicity" and do not take into account the broad effects of hyperglycemia on skin vascularity, immune response etc. Thus, *in-vitro* and *ex-vivo* diabetic wound models show clear limitations but can provide a first hint for the putative effectivity of an active ingredient which has to be further investigated *in-vivo*. A further advancement may be the use of *ex-vivo* diabetic skin, if accessibility is not a problem. This should be tested in a future study.

## Supporting Information

S1 Fig Comparison of data gained with classical scratch assay and semi-automated system. Closed scratch wound area per visual field in classical scratch assay (A, C, E, G) and semi-automated scratch assay (B, D, F, H) of diabetic/non-diabetic keratinocytes (A, B), adult non-diabetic keratinocytes under eu- and hyperglycaemic conditions (C, D) and adult non-diabetic keratinocytes under euglycaemic (E, F) and hyperglycaemic (G, H) conditions treated with DMSO (1:1000 in medium), TE (1 μg/ml) or betulin (0.87 μg/ml) (E-H) Mean ± SEM; *: statistically significant with p < 0.05.
(DOCX)

S2 Fig Influence of 6 mM and 25 mM glucose with and without preincubation on reepithelialization in ex-vivo wound healing models. Length of the regenerated epidermis of porcine *ex-vivo* models cultured in medium containing 6 mM or 25 mM glucose for 48 hours after wounding with and without preincubation for 48 h (n = 10 in triplicates; mean ± SEM).
(DOCX)

S3 Fig Influence of TE and betulin on scratch wound healing under various conditions. Closed scratch wound area per visual field of human primary keratinocytes from juvenile (A, B) as well as adult non-diabetic (C, D) and adult diabetic (E, F) donors, treated with DMSO (1:1000 in medium), TE (1 μg/ml) or betulin (0.87 μg/ml), under (A, C, E) euglycaemic (6 mM glucose) and (B, D, F) hyperglycaemic (25 mM glucose) conditions at the indicated time points after wounding (n = 4 in duplicates); mean ± SEM; *: statistically significant with p < 0.05.
(DOCX)

S4 Fig Influence of TE and betulin (lower concentrations) on scratch wound healing of normal keratinocytes under eu- and hyperglycaemic conditions. Closed scratch wound area per visual field of human primary keratinocytes from adult, non-diabetic donors, that were treated with DMSO (1:10000), TE (100 ng/ml) or betulin (87 ng/ml) under (A, C) euglycaemic (6 mM) and (B, D) hyperglycaemic (25 mM) conditions at 4, 8, 12, 24 and 36 hours after wounding. (A, B) conventional scratch assay and (C, D) semi-automatic system. (n = 4 in duplicates for the conventional scratch assay and n = 3 at least in duplicates for the semi-automatic system; mean ± SEM).
(DOCX)

S5 Fig Influence of TE and betulin (lower concentrations) on scratch wound healing of diabetic keratinocytes under eu- and hyperglycaemic conditions. Closed scratch wound area per visual field of human primary keratinocytes from adult, diabetic donors, that were treated with DMSO (1:10000), TE (100 ng/ml) or betulin (87 ng/ml), under (A) euglycaemic (6 mM) and (B) hyperglycaemic (25 mM) conditions at 4, 8, 12, 24 and 36 hours after wounding. (n = 4 at least in duplicates; mean ± SEM).
(DOCX)

S6 Fig Influence of PDGF, TE and betulin on scratch wound healing of non-diabetic fibroblasts. Closed scratch wound area as % of control 12 hours after wounding. (n = 4 mean ± SD).
(DOCX)

S1 File Description of the two scratch assay systems and discussion of the differences, advantages and limitations. (DOCX)

S2 File Influence of TE on *in-vitro* models in diabetic and non-diabetic cells under eu- and hyperglycaemic conditions. (DOCX)

S1 Table Comparison of important parameters of the conventional and the semi-automated scratch assay. (DOCX)

S2 Table Overview of the statistical parameters used for statistical evaluation of the various *in-vitro* WH settings. (DOCX)
