# Supplementary material for: Comparison of In-Vitro and Ex-Vivo Wound Healing Assays for the Investigation of Diabetic Wound Healing and Demonstration of a Beneficial Effect of a Triterpene Extract
Source: PLoS One. 2017 Jan 3;12(1):e0169028. doi: 10.1371/journal.pone.0169028 (PMC5207624; doi:10.1371/journal.pone.0169028)
Supplement: S1 Fig — Closed scratch wound area per visual field in classical scratch assay (A, C, E, G) and semi-automated scratch assay (B, D, F, H) of diabetic/non-diabetic keratinocytes (A, B), adult non-diabetic keratinocytes under eu- and hyperglycaemic conditions (C, D) and adult non-diabetic keratinocytes under euglycaemic (E, F) and hyperglycaemic (G, H) conditions treated with DMSO (1:1000 in medium), TE (1 μg/ml) or betulin (0.87 μg/ml) (E-H) Mean ± SEM; *: statistically significant with p < 0.05. (DOCX) [file pone.0169028.s001.docx]

**Supplemental Figure 1**


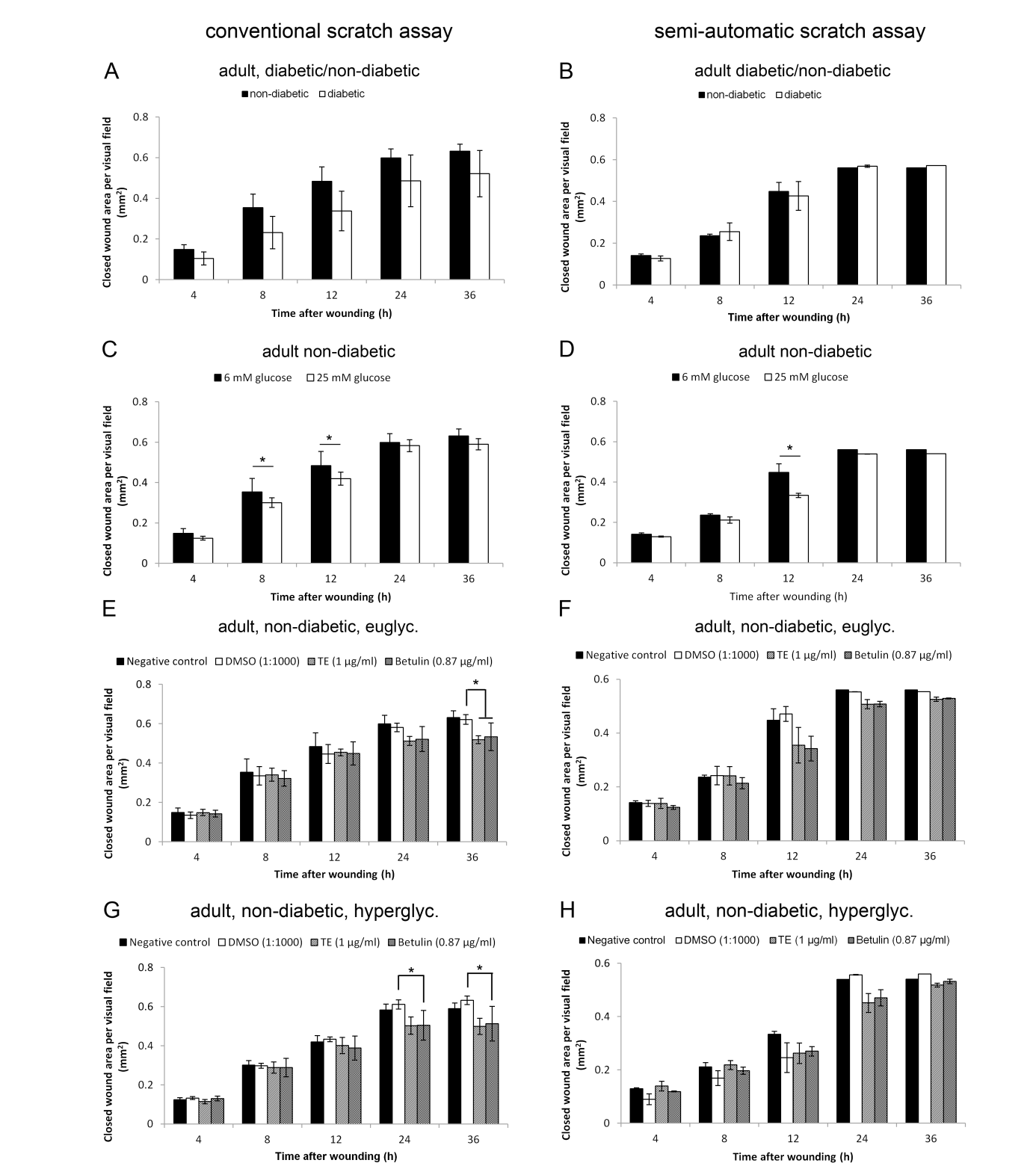


**S1 Fig:** **Comparison of data gained with classical scratch assay and semi-automated system.** Closed scratch wound area per visual field in classical scratch assay (A, C, E, G) and semi-automated scratch assay (B, D, F, H) of diabetic/non-diabetic keratinocytes (A,B), adult non-diabetic keratinocytes under eu- and hyperglycaemic conditions (C, D) and adult non-diabetic keratinocytes under euglycaemic (E, F) and hyperglycaemic (G, H) conditions treated with DMSO (1:1000 in medium), TE (1 µg/ml) or betulin (0.87 µg/ml) (E-H) Mean ± SEM; *: statistically significant with p < 0.05.
