# Supplementary material for: Comparison of In-Vitro and Ex-Vivo Wound Healing Assays for the Investigation of Diabetic Wound Healing and Demonstration of a Beneficial Effect of a Triterpene Extract
Source: PLoS One. 2017 Jan 3;12(1):e0169028. doi: 10.1371/journal.pone.0169028 (PMC5207624; doi:10.1371/journal.pone.0169028)
Supplement: S2 Fig — Length of the regenerated epidermis of porcine ex-vivo models cultured in medium containing 6 mM or 25 mM glucose for 48 hours after wounding with and without preincubation for 48 h (n = 10 in triplicates; mean ± SEM). (DOCX) [file pone.0169028.s002.docx]

**Supplemental Figure 2**

**

**

**S2 Fig. Influence of 6 mM and 25 mM glucose with and without preincubation on reepithelialization in ex-vivo wound healing models.** Length of the regenerated epidermis of porcine ex-vivo models cultured in medium containing 6 mM or 25 mM glucose for 48 hours after wounding with and without preincubation for 48 h (n = 10 in triplicates; mean ± SEM).
