# Supplementary material for: Comparison of In-Vitro and Ex-Vivo Wound Healing Assays for the Investigation of Diabetic Wound Healing and Demonstration of a Beneficial Effect of a Triterpene Extract
Source: PLoS One. 2017 Jan 3;12(1):e0169028. doi: 10.1371/journal.pone.0169028 (PMC5207624; doi:10.1371/journal.pone.0169028)
Supplement: S3 Fig — Closed scratch wound area per visual field of human primary keratinocytes from juvenile (A, B) as well as adult non-diabetic (C, D) and adult diabetic (E, F) donors, treated with DMSO (1:1000 in medium), TE (1 μg/ml) or betulin (0.87 μg/ml), under (A, C, E) euglycaemic (6 mM glucose) and (B, D, F) hyperglycaemic (25 mM glucose) conditions at the indicated time points after wounding (n = 4 in duplicates); mean ± SEM; *: statistically significant with p < 0.05. (DOCX) [file pone.0169028.s003.docx]

**Supplemental Figure 3**

**
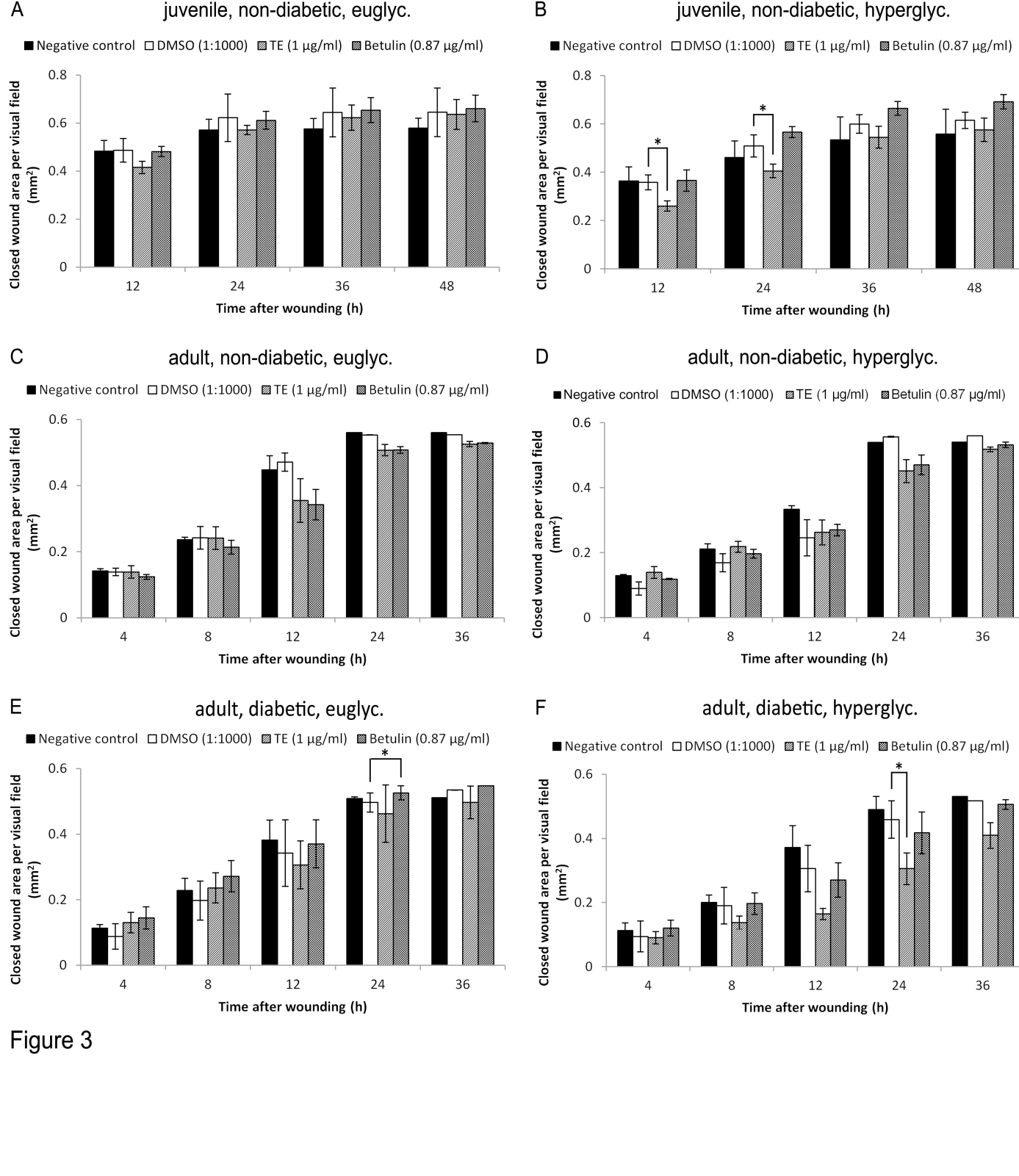
**

**S3 Fig. Influence of TE and betulin on scratch wound healing under various conditions.** Closed scratch wound area per visual field of human primary keratinocytes from juvenile (A, B) as well as adult non-diabetic (C, D) and adult diabetic (E, F) donors, treated with DMSO (1:1000 in medium), TE (1 µg/ml) or betulin (0.87 µg/ml), under (A, C, E) euglycaemic (6 mM glucose) and (B, D, F) hyperglycaemic (25 mM glucose) conditions at the indicated time points after wounding (n = 4 in duplicates); Mean ± SEM; *: statistically significant with p < 0.05.
