# Supplementary material for: Comparison of In-Vitro and Ex-Vivo Wound Healing Assays for the Investigation of Diabetic Wound Healing and Demonstration of a Beneficial Effect of a Triterpene Extract
Source: PLoS One. 2017 Jan 3;12(1):e0169028. doi: 10.1371/journal.pone.0169028 (PMC5207624; doi:10.1371/journal.pone.0169028)
Supplement: S4 Fig — Closed scratch wound area per visual field of human primary keratinocytes from adult, non-diabetic donors, that were treated with DMSO (1:10000), TE (100 ng/ml) or betulin (87 ng/ml) under (A, C) euglycaemic (6 mM) and (B, D) hyperglycaemic (25 mM) conditions at 4, 8, 12, 24 and 36 hours after wounding. (A, B) conventional scratch assay and (C, D) semi-automatic system. (n = 4 in duplicates for the conventional scratch assay and n = 3 at least in duplicates for the semi-automatic system; mean ± SEM). (DOCX) [file pone.0169028.s004.docx]

**Supplemental Figure 4**

**
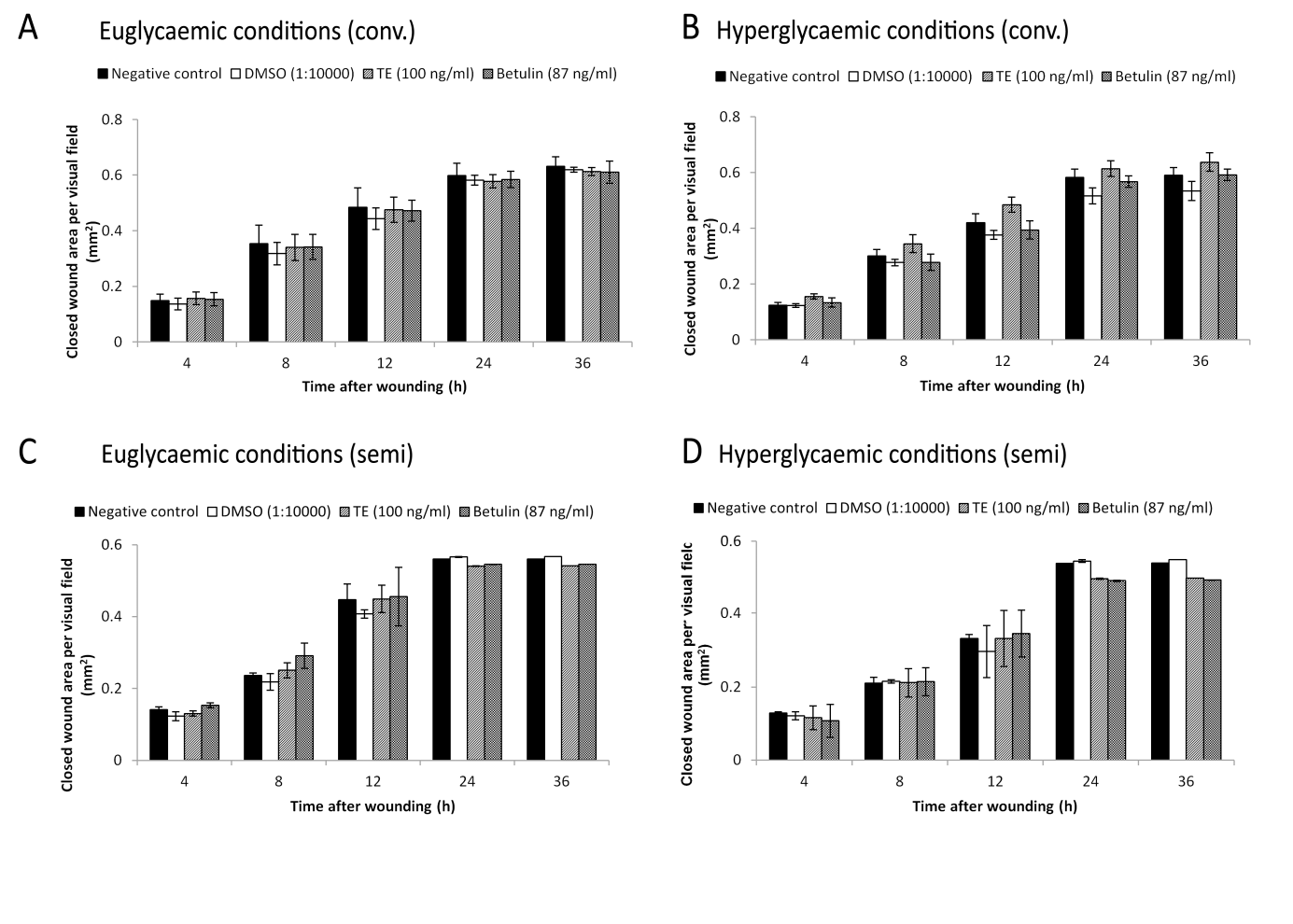
**

**S4 Fig. Influence of TE and betulin (lower concentrations) on scratch wound healing of normal keratinocytes under eu- and hyperglycaemic conditions.** Closed scratch wound area per visual field of human primary keratinocytes from adult, non-diabetic donors, that were treated with DMSO (1:10000), TE (100 ng/ml) or betulin (87 ng/ml) under (**A**, **C**) euglycaemic (6 mM) and (**B**, **D**) hyperglycaemic (25 mM) conditions at 4, 8, 12, 24 and 36 hours after wounding. (**A, B**) conventional scratch assay and (**C, D**) semi-automatic system. (n = 4 in duplicates for the conventional scratch assay and n = 3 at least in duplicates for the semi-automatic system; mean ± SEM).
