# Supplementary material for: Comparison of In-Vitro and Ex-Vivo Wound Healing Assays for the Investigation of Diabetic Wound Healing and Demonstration of a Beneficial Effect of a Triterpene Extract
Source: PLoS One. 2017 Jan 3;12(1):e0169028. doi: 10.1371/journal.pone.0169028 (PMC5207624; doi:10.1371/journal.pone.0169028)
Supplement: S5 Fig — Closed scratch wound area per visual field of human primary keratinocytes from adult, diabetic donors, that were treated with DMSO (1:10000), TE (100 ng/ml) or betulin (87 ng/ml), under (A) euglycaemic (6 mM) and (B) hyperglycaemic (25 mM) conditions at 4, 8, 12, 24 and 36 hours after wounding. (n = 4 at least in duplicates; mean ± SEM). (DOCX) [file pone.0169028.s005.docx]

**Supplemental Figure 5**


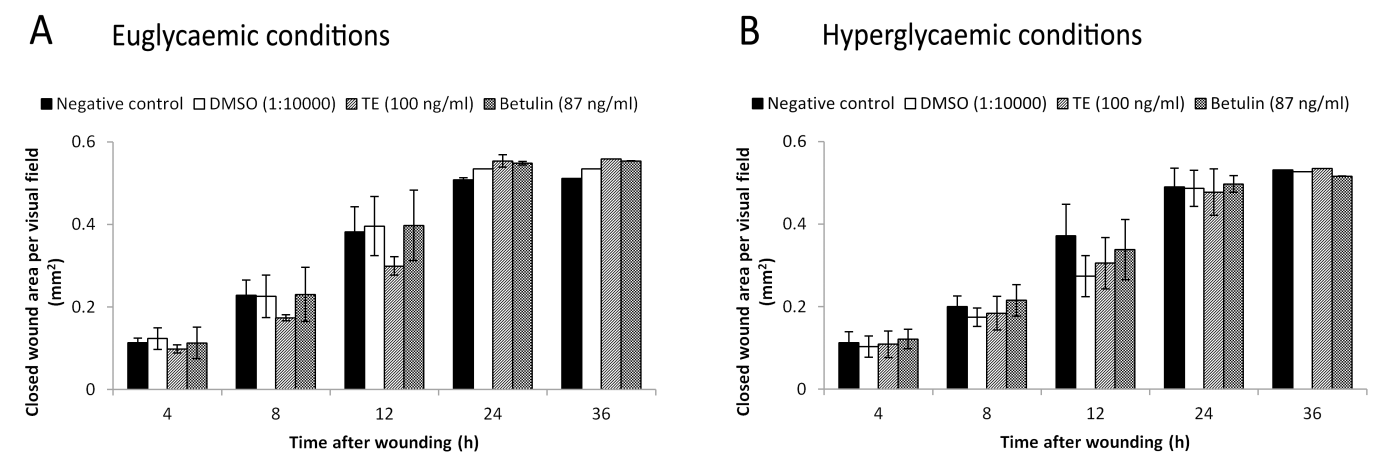


**S5 Fig. Influence of TE and betulin (lower concentrations) on scratch wound healing of diabetic keratinocytes under eu- and hyperglycaemic conditions.** Closed scratch wound area per visual field of human primary keratinocytes from adult, diabetic donors, that were treated with DMSO (1:10000), TE (100 ng/ml) or betulin (87 ng/ml), under (**A**) euglycaemic (6 mM) and (**B**) hyperglycaemic (25 mM) conditions at 4, 8, 12, 24 and 36 hours after wounding. (n = 4 at least in duplicates; mean ± SEM).
