# Supplementary material for: Comparison of In-Vitro and Ex-Vivo Wound Healing Assays for the Investigation of Diabetic Wound Healing and Demonstration of a Beneficial Effect of a Triterpene Extract
Source: PLoS One. 2017 Jan 3;12(1):e0169028. doi: 10.1371/journal.pone.0169028 (PMC5207624; doi:10.1371/journal.pone.0169028)
Supplement: S6 Fig — Closed scratch wound area as % of control 12 hours after wounding. (n = 4 mean ± SD). (DOCX) [file pone.0169028.s006.docx]

**Supplemental Figure 6**

*

**S6 Fig. Influence of PDGF, TE and betulin on scratch wound healing of non-diabetic fibroblasts.** Closed scratch wound area as % of control 12 hours after wounding. (n = 4 mean ± SD).
