# Supplementary material for: Comparison of In-Vitro and Ex-Vivo Wound Healing Assays for the Investigation of Diabetic Wound Healing and Demonstration of a Beneficial Effect of a Triterpene Extract
Source: PLoS One. 2017 Jan 3;12(1):e0169028. doi: 10.1371/journal.pone.0169028 (PMC5207624; doi:10.1371/journal.pone.0169028)
Supplement: S1 File — (DOCX) [file pone.0169028.s007.docx]

S1 File: Supplemental data

**Comparison of the two scratch assay test systems:**

In this manuscript two different scratch assay test systems were used. On one hand the conventional scratch assay, on the other hand a semi-automated (high-throughput) system (Essen BioScience, LTd., Hertfordshire, United Kingdom). For a comparison of technical data of the two systems see S1 Table.

Both methods were used to investigate diabetic cells versus non-diabetic cells (S1A, B Fig), adult non-diabetic cells under eu- and hyperglycaemic conditions (S1C, D Fig), and adult non-diabetic cells under euglycaemic (S1E, F Fig, S4A, C Fig) and hyperglycaemic (S1G, H Fig, S4B, D Fig) conditions after treatment with TE and betulin. An important advantage of the semi-automated system is the 96-well format which allows parallel screening of many samples and which reduces the amount of cells needed. In addition, a higher frequency of evaluated time points is possible, because the pictures are taken continuously without the necessity to remove the cultures from the incubator. Both methods showed largely similar results. Differences between experimental groups were sometimes more pronounced – but only moderately - with the conventional system. One explanation might be that disturbance of the cell culture due to the removal from the incubator in the conventional scratch assay may intensify small differences.
