# Supplementary material for: Comparison of In-Vitro and Ex-Vivo Wound Healing Assays for the Investigation of Diabetic Wound Healing and Demonstration of a Beneficial Effect of a Triterpene Extract
Source: PLoS One. 2017 Jan 3;12(1):e0169028. doi: 10.1371/journal.pone.0169028 (PMC5207624; doi:10.1371/journal.pone.0169028)
Supplement: S2 File — (DOCX) [file pone.0169028.s008.docx]

S2 File: Supplemental data

**Influence of TE on *in-vitro* models**

Treating juvenile or adult non-diabetic keratinocytes with 1 µg/ml TE or 0.87 µg/ml betulin (concentration of betulin in 1 µg/ml TE) did not result in a positively altered scratch wound closure under euglycaemic conditions (S3A. C Fig). When using 100 ng/ml TE and the respective concentration of betulin (87 ng/ml), there was also no significant effect on adult keratinocytes under euglycaemic conditions (S4 Fig). Furthermore, there was also no positive effect with higher concentrations of TE (5 µg/ml, 10 µg/ml; data not shown). In diabetic keratinocytes under euglycaemic conditions, TE displayed no significant effect, while betulin exhibited a slight but significantly positive effect 24 h after scratch wounding (from 0.50 ± 0.02 mm^2^ to 0.53 ± 0.02 mm^2^, 106 ± 4.4 % of control) (S3E Fig). Lower concentrations of TE and betulin did not result in significant alterations in diabetic keratinocytes (S5 Fig).

Under hyperglycaemic conditions, a significant negative effect of 1 µg/ml TE (p<0.05) was observed 12 h (closed wound area with TE 0.26 ± 0.02 mm^2^ compared to 0.36 ± 0.02 mm^2^ in DMSO controls, i.e. 73 ± 6 % of control) and 24 h (closed wound area with TE 0.41 ± 0.03 mm^2^ compared to 0.51 mm^2^ ± 0.03 in DMSO controls , i.e. 80 ± 6 % of control) after wounding in juvenile keratinocytes (S3B Fig), which was not the case in adult hyperglycaemic cells (S3D Fig). For diabetic cells under hyperglycaemic conditions, there was a significantly reduced scratch closure for TE treatment at 24 h (0.31 ± 0.05 mm^2^ in TE treated cells compared to 0.46 ± 0.06 mm^2^ in DMSO control cells, that means 67 ± 11 % of control) (S3F Fig*)*. Lower concentrations of TE and betulin did not lead to significant alterations (S5 Fig).

Summarizing these data, the choice of cells (young, adult, diabetic, non-diabetic) influenced the outcome of the experiment. In addition, the concentration of TE and time point of evaluation was of importance. However, in general, no positive effect was observed for TE.
