# Supplementary material for: Comparison of In-Vitro and Ex-Vivo Wound Healing Assays for the Investigation of Diabetic Wound Healing and Demonstration of a Beneficial Effect of a Triterpene Extract
Source: PLoS One. 2017 Jan 3;12(1):e0169028. doi: 10.1371/journal.pone.0169028 (PMC5207624; doi:10.1371/journal.pone.0169028)
Supplement: S1 Table — (DOCX) [file pone.0169028.s009.docx]

**Supplemental Table 1**

**S1 Table: Comparison of important parameters of the conventional and the semi-automated scratch assay.**

| **Assay Type**  **Parameters** | **Conventional scratch assay** | **Semi-automated system** |
| --- | --- | --- |
| Well format | 12-well | 96-well |
| Amount of cells at seeding (48h before scratch) | 200,000 cells (infant keratinocytes)  250,000 cells (adult keratinocytes) | 25,000 cells (adult keratinocytes) |
| Density at scratch formation | 100% | 100% |
| Method for scratch generation | Removal of cells in central area by a sterile pipette tip (10-200 µl), washing with PBS, supply with fresh medium | Removal of cells in central area by the Woundmaker^TM^ tool (Essen BioScience, Ltd), washing with PBS, supply with fresh medium |
| Scratch width | 0.65 mm ± 0.088 mm | 0.65 mm ± 0.066 mm |
| Total scratch size/total well size | 3.8% +/- 0.5% | 13% +/- 1.3% |
| Culture conditions | 37°C, 5% CO_2_, saturated humidity, removal from the incubator for photography | 37°C, 5% CO_2_, saturated humidity, no removal from the incubator for photography |
| Pictures per well | at least 3 | 1 |
| Frequency of pictures | Every 4 or 12 h up to 36 h | Every h, up to 36 h |
| Technical replicates | Duplicates | At least duplicates |
| Kind of migration | Keratinocyte sheet | Keratinocyte sheet |
