# Supplementary material for: Comparison of In-Vitro and Ex-Vivo Wound Healing Assays for the Investigation of Diabetic Wound Healing and Demonstration of a Beneficial Effect of a Triterpene Extract
Source: PLoS One. 2017 Jan 3;12(1):e0169028. doi: 10.1371/journal.pone.0169028 (PMC5207624; doi:10.1371/journal.pone.0169028)
Supplement: S2 Table — (DOCX) [file pone.0169028.s010.docx]

**Supplemental Table 2**

**S2 Table –** Overview of the statistical parameters used for statistical evaluation of the various in-vitro settings

| **In vitro setting** | **Main effect** | **Interaction term with** | **Additional predictor/**  **confounder** | **Dependent variable** |
| --- | --- | --- | --- | --- |
| Juvenile keratinocytes | - TE/Betulin treatment - Supplement - Media | Glucose/mannitol concentration | Time/ baseline scratch wound size | Change of the scratch wound size |
| Adult keratinocytes  Conventional scratch assay | Diabetic vs nondiabetic donor | Time | Baseline scratch wound size | Change of the scratch wound size |
| Adult keratinocytes (Subgroup non-diabetic donor)  Conventional scratch assay | TE/Betulin treatment | Glucose concentration and time | Baseline scratch wound size | Change of the scratch wound size |
| Adult keratinocytes  Semiautomated system | Diabetic vs nondiabetic donor for TE/Betulin treatment | Glucose concentration and time | Baseline scratch wound size | Change of the scratch wound size |
| Adult vs juvenile keratinocytes | Adult vs juvenile cells | Glucose concentration and time | Baseline scratch wound size | Change of the scratch wound size |
